# Characterization of Bioactive Compounds, Mineral Content and Antioxidant Capacity in Bean Varieties Grown in Semi-Arid Conditions in Zacatecas, Mexico

**DOI:** 10.3390/foods7120199

**Published:** 2018-12-05

**Authors:** Ibeth Marlene Herrera-Hernández, Karen Vanesa Armendáriz-Fernández, Ezequiel Muñoz-Márquez, Juan Pedro Sida-Arreola, Esteban Sánchez

**Affiliations:** 1 Unidad Académica Meoqui, Universidad Tecnológica de Camargo, De Zaragoza 1517, Deportiva, Pedro Meoqui 33130, Mexico; marleneherrera.ih@gmail.com (I.M.H.-H.); karenvane150@gmail.com (K.V.A.-F.); 2 Delicias Unit, Centro de Investigación en Alimentación y Desarrollo (Center for Food and Development Research), Delicias, Chihuahua 33089, Mexico; emunoz@ciad.mx (E.M.-M.); juan.sida@ciad.mx (J.P.S.-A.)

**Keywords:** common bean (*Phaseolus vulgaris* L.), biofortification, antioxidant capacity, bioactive compounds, mineral content

## Abstract

This research describes the characterization of bioactive compounds, mineral content, and antioxidant capacity in bean varieties grown in semi-arid conditions in Zacatecas, Mexico. This will provide better progress perspectives for agriculture nationwide and will ensure that bean crops are in the condition to satisfy the population’s nutritional needs by placing it not only as one of the foodstuffs comprising Mexico’s basic diet but also as one of the legumes having greater biofortification potential in Mexico. Eleven bean varieties were collected (flor de mayo, bayo, frijola, patola, navy beans, flor de junio, reata beans, Japanese beans, black beans, canary beans, and pinto Saltillo). The evaluation done included the physical and physico-chemical characteristics, as well as the mineral content, bioactive compounds and antioxidant capacity of these bean varieties. Data gathered were subject to a variance and mean separation analysis. The most remarkable individual results were as follows: Canary beans had the highest iron content (105.29 mg/kg), while bayo beans had the highest zinc concentration (48.18 mg/kg) and reata beans had the highest level of protein (26.88%). The varieties showing the most remarkable results with regard to zinc, iron and protein content and antioxidant capacity were as follows: Flor de junio, flor de mayo, reata beans, navy beans and pinto Saltillo; however, the most remarkable variety in comprehensive terms was flor de junio, which showed a reducing power of 0.20%, an antioxidant capacity of 80.62% inhibition, a protein content of 0.17%, in addition to Ca (0.24%), Fe (90.97 mg/kg), Zn (27.23 mg/kg), fiber (3.22%), energy (353.50 kcal), phenols (94.82 mg gallic acid (GA)/g extract) and flavonoids (1.30 mg mg Cat/g^−1^ dw). Finally, we came to the conclusion that beans grown in Zacatecas, Mexico, offer a huge benefit to consumers as a result of the mineral, protein, fiber, bioactive compounds, and antioxidant capacity contributions such beans provide. Thus, these beans can be used in a biofortification program using micronutrients to improve their nutritional quality.

## 1. Introduction

Micronutrient deficiency in horticultural crops has increased in recent years, resulting in a marked decrease in the quality of people’s diet in almost all the world. Deficiency in micronutrients such as iodine, selenium iron, and zinc has even been considered a public health problem which affects more than one third of the world’s population, resulting in an increase in mortality rates, shorter heights and learning problems [1]. However, there is currently a growing interest in nutrition and eating habits. This has caused individuals to be more selective regarding the products they eat. Vegetable-based diets have raised people’s interest since such diets contain the main nutrients required by the body to perform its various functions and cover people’s dietary needs. Hence, vegetable origin products are now being considered as foodstuffs having a high nutraceutical potential. One such product is beans, an important legume around the world in terms of human consumption. However, both producers and consumers are unaware of the exact nutritional value deriving from this preserved genetic diversity, even though it forms part of their daily diet [2]. Common bean (*Phaseolus vulgaris* L.) is considered to be the most important legume in the world. It is a major source of protein, calories, B-complex vitamins, minerals, polyphenols and other elements which collectively give beans a high nutraceutical value [3,4]. Given that beans are readily available, have a relatively low price and are associated with cultural tradition, they have been classified as one of the pillars supporting ancient gastronomic culture and as an indispensable product forming part of the consumer basket. This legume incorporates certain characteristics that help complete a nutrition table [5]. Beans represent a considerable source of micronutrients, such as iron, calcium, phosphorus, magnesium and zinc. Beans contain a good level of bioactive compounds. These substances can influence human health, having an impact on various activities at the physiological or cellular level. Beans improve our health, counteract the aging process, prevent chronic diseases, extend life expectancy and support the bodily structure or function [6]. The human body’s inability to neutralize free radicals, individuals must resort to foods that are able to neutralize them. Beans provide beneficial levels of antioxidant capacity and substances that are able to prevent or slow down oxidation in easily oxidized materials. Beans protect the body against the action of free radicals by slowing down the aging process and fighting cell degeneration and cell death [7]. 

Mexico is considered to be the country possessing the greatest variety of beans, since 47 of the 52 *Phaseolus* species originated in Mexico [8]. In 2017, domestic production amounted to 1,273,957 metric tons, which represents a sown surface of 1,428,000 hectares (3,528,665 acres) [9]. In recent years, the state of Zacatecas has become the largest bean producer nationwide, having an annual production from 350,000 to 450,000 metric tons [5]. Given that characterization is a step prior to the implementation of biofortification programs, improving crops by using fertilization techniques, traditional plant breeding or biotechnology assisted breeding is considered a viable alternative to increase nutrient content. However, studies on the physico-chemical properties, bioactive compounds and minerals content, as well as the antioxidant capacity of the bean varieties grown and produced in Mexico have yet to be carried out. The main purpose of this article is to provide the nutritional characterization of the bean varieties grown in Zacatecas, Mexico. This has the potential to implement agronomic biofortification models that serve as a feasible alternative for nutritional improvement of the population, especially in poor communities living in Mexico’s urban and rural areas. This will provide better progress perspectives for agriculture nationwide and will ensure that bean crops are in the condition to satisfy the population’s nutritional needs by placing it not only as one of the foodstuffs comprising Mexico’s basic diet, but also as one of the legumes having greater biofortification potential in Mexico.

## 2. Materials and Methods

### 2.1. Plant Material

Eleven varieties of bean (*Phaseolus vulgaris* L.) were collected in the state of Zacatecas, Mexico (Table 1).

### 2.2. Sample Preparation.

One-hundred seeds were taken per bean variety, which were then grounded using a blender. The result of each grinding was placed in aseptic bags. These bags were later used in the analysis.

### 2.3. Plant Analysis

A total of 32 variables were analyzed in 11 bean varieties from the Calera region (state of Zacatecas, Mexico). The variables analyzed were as follows: physical properties (length, width, thickness, weight, L*, a*, b* and °hue); physico-chemical properties (ash, fat, moisture, fiber, carbohydrates, protein and energy); mineral analysis (Fe, Zn, Ca, Mg, K, Ni, Na, Cu, Mn, P and N); bioactive compounds (total phenols, flavonoids and anthocyanins); antioxidant capacity and reducing power.

#### 2.3.1. Physical Properties

##### Seed Dimensions

The length, width and thickness parameters were determined using an electronic Vernier caliper in order to test 40 seeds per bean variety studied. The purpose of this was to obtain a comparison in both qualitative terms and quantitative terms with regard to the differences in size among bean varieties. This resulted in a total of 440 data points per parameter, which are reported in millimeters (mm) [10].

##### Seed Weight

Weight was determine using an analytical balance. One-hundred seeds were placed in a Petri dish representing each bean variety used in the study. The Petri dish containing the seeds was then placed on the analytical balance. This procedure was performed in triplicate. Weight was reported in g/100 seeds [11].

##### Seed Color

A colorimeter was used to determine color using a Minolta CM-2600d spectrophotometer (Konica Minolta Sensing Inc., Osaka, Japan) with an observed D65 illumination at the 10° angle. Measurements were performed in triplicate on each bean variety used in this study. Afterwards, the mean of these measurements was obtained. Parameters L*, a*, b* were recorded. Then, chromaticity and the ($C^{*}=\sqrt{{\left(a^{*}\right)}^{2}+{\left(b^{*}\right)}^{2}}$) hue angle (h° = tan − 1(b*/a*)) were calculated for each bean variety studied. Results were analyzed to finally obtain color saturation. Where L* is luminance, taking the 100 (white) and 0 (black) values as reference, while a* and b* have no limits, though they include positive and negative values. The a* scale varies, showing both positive (red) and negative (green) values, whereas the b* scale varies from yellow (+) to blue (-).

#### 2.3.2. Physico-Chemical Properties

##### Moisture Determination

This was done using the open dish drying method established by the Association of Official Analytical Chemists [12]. The following procedure was done in triplicate: 1 g of each bean variety studied was weighed in an aluminum dish previously dried to constant weight at 75 °C. After having weighed each dish, dishes were introduced in a Felisa oven at 75 °C for 12 hours. After this period elapsed, dishes were taken out of the oven, leaving them to dry in a desiccator, after which they were weighed once again. Moisture content was determined based on the weight change as a percentage.

##### Ash Determination

This measurement was done in accordance with Mexican Standard NMX-F-066-S-1978 [13] and Harris and Marshall [14]. Using a crucible, 1 g of the sample was weighed to constant weight (two repetitions per variety). Upon weighing, samples were placed in a desiccator. Then, samples were placed in crucibles and introduced to a muffle (Felisa) at 600 °C for 24 h in order to carbonize the samples until charring. Crucibles were removed from the muffle and placed into a desiccator. Crucible weight was performed 24 h after they were placed into the desiccator. Percentage of ash was estimated with the following formula:

$$\%\;ash=\frac{\left(P-p\right)\times 100}{M}$$

where:

P = mass of the crucible with ash in grams;
p = mass of the empty crucible in grams;
M = mass of the sample in grams.

##### Fat Determination

This measurement was done using the Goldfish method [15]. Goldfish flasks were prepared by drying them to constant weight using a heater. The LABCONCO equipment (Labconco, Kansas City, MO, USA) was then installed. Samples were placed in filter paper, covering them with cotton and then placing them inside the equipment. Petroleum ether was added as solvent, leaving it in reflux for 2 h and 30 min. Upon extraction completion, the solvent was recovered by distillation, leaving only the fat in the flask. Finally, each flask containing this residue was weighed as described in Mexican Standard NMX-F-427-1982 [16], determining the percentage of fat.

##### Fiber Determination

The crude fiber concentration was measured according to Mexican Standard NMX-F-90-S-1978 [17]. This determination was done using the previously degreased sample. Samples were weighed, recording each weight. Then, each sample was transferred to fiber cups, adding 200 mL of 1.25% sulfuric acid and 1 mL of isoamyl alcohol (antifoam agent) to each cup. The resulting mixture was allowed to boil for 30 min, after which samples were subject to fiberglass rinsing until neutralized. Afterwards, the fiberglass containing the sample was placed in a dish and introduced in a heater, where it was allowed to dry for 12 h to ensure the sample was perfectly dry. After this period, the dish and fiberglass containing the sample were weighed. The fiber percentage contained in each sample was determined as the difference in such weights.

##### Carbohydrate Determination

This measurement was done by calculating the difference in other parameters and is shown as a percentage [18].

##### Energy Determination

Energy contained in each sample was determined by the sum of calories contained in carbohydrates, fats and protein as described in the Mexican Standard NOM-051-SCFI/SSA1-2010. Energy was expressed in kcal/100g.

#### 3.3.3. Mineral Analysis

##### Microelements

For the micronutrients analysis, one-hundred seeds were taken per bean variety, which were then grounded using a blender. The result of each grinding was placed in aseptic bags. After this, 1 g of sample was weighed using an analytical balance, starting with digestion procedures on a digestion plate. Digests were done in triplicate for each bean variety by weighing 1 g of the sample. The entire process was done inside a flow cabinet where the sample was placed over a beaker containing 3 boiling stones, adding 25 mL of triacid solution (nitric acid, hydrochloric acid and sulfuric acid). After this, samples were covered and placed on the plate at 3-level fire, thus starting the digestion process, which lasted approximately one hour.

After this period elapsed, samples were removed from the fire, allowing them to cool down for a few minutes. While they were cooling down, a glass funnel was placed in a 50 mL volumetric flask. Filter paper was used to make a paper cone, placing it over the funnel. Once samples were cold, triple distilled water was sprayed over the beaker to rinse the digest, which was poured into the flask. Then, two additional rinse procedures were performed. Upon completing the third rinse procedure, the filter paper and funnel were removed. Using a wash bottle containing triple distilled water, the flask content was diluted to a volume of 50 mL. Finally, samples were poured in 50 mL tubes (previously labeled to facilitate identification). A total of 33 tubes were used for the 11 bean varieties studied. 

Upon completion of the digestion process, the concentrations of the micronutrients contained in each of the 11 bean varieties were measured. For this, samples were read using the atomic absorption device (AAS, iCE 3000 Series, Thermo Scientific^®^, Franklin, MA, USA), which had to be adjusted when measuring each element. This adjustment consisted in changing the lamps in the device depending on the element and programing the device using a computer. Results are shown in parts per million (mg kg^−1^).

##### Macroelements

To determine the concentration of macronutrients, the same method for micronutrients was applied using the atomic absorption device (AAS, iCE 3000 Series, Thermo Scientific^®^). Results are shown as percentages (%).

Phosphorus (P) content was determined by means of the ammonium metavanadate (NH_4_VO_3_) method using an absorption range of 430 nm against a K_2_HPO_4_ pattern curve. To prepare the phosphorus reagent, we used a beaker containing 800 mL of hot deionized water (near boiling point) to dissolve 10 g of ammonium molybdate [(NH_4_)_6_Mo_7_O_24_·4H_2_O] and 0.5 g of ammonium vanadate [NH_4_VO_3_]. Afterwards, when the solution was already called, 4 mL of HNO_3_ were added (drop-wise in the beginning), stirring continuously. Then, 134 mL of HNO_3_ were added. The solution was diluted to a final volume of 1 L with deionized water. Then, 3.5 mL of triple distilled water were transferred to test tubes (2 tubes per repetition with each sample), adding 500 μL of sample of the previously digested variety. Finally, 1 mL of phosphorus reagent was added to each test tube, mixing the tubes in a Vortex mixer (VWR) and letting them rest for an hour. After this one-hour period, we took the reading of each sample using a visible light spectrophotometry device (Jenway 6405 UV-vis, Jenway, Stafforshire, UK), pouring the resulting liquid in the cells and placing the cells in the spectrophotometer at 430 nm. The phosphorus concentration is shown as a percentage.

#### 2.3.4. Antioxidant Capacity

This analysis was done based on the methodology used by Brand Williams et al. [19]. The extract was obtained homogenizing 1 g of seeds in 5 mL of 80% methanol. The resulting mixture was centrifuged at 2560× *g* for 10 min at 4 °C, taking 0.5 mL of the resulting supernatant from extract to mix it with 2.5 mL of 0.1 mM 2,2-diphenyl-1-picrylhydrazyl (DPPH) solution. The freshly prepared mixture was incubated for 60 min in darkness and cold conditions. Absorbance was measured at 517 nm using a spectrophotometer (Jenway 6405). The antioxidant capacity is expressed as percentage of ability to quench DPPH radicals and estimated with the following equation:

$$\%\;\text{ability to quench DPPH radicals}=\left(1-\frac{A_{\mathrm{sample}}-A_{\mathrm{blank}}}{A_{\mathrm{reference}}}\right)\times 100$$

where:

A_sample_ = Absorbance of the sample;
A_blank_ = Absorbance of the blank;
A_reference_ = Absorbance of the reference containing the radical DPPH.

#### 2.3.5. Reducing Power

The reducing power of seeds was measured based on the method used by Oyaizu [20]. The extract was obtained by soaking in ice 1 g of seeds in 5 mL of 80% methanol. This was centrifuged at 1775× *g* for 10 min at 4 °C. From the resulting supernatant, 1 mL of extract was taken, adding 1 mL of 0.2 M (pH = 6.6) phosphate buffer and 1 mL of 1% K_3_Fe(CN)_6_ (*w*/*v*). Then, the mixture was incubated for 20 min at 50 °C. Then, test tubes were cooled down by immersing them in ice for 10 min, after which 0.5 mL of 10% CL_3_CCOOH was added to the test tubes. After this 10-min period, test tubes were centrifuged at 1775× *g* for 10 min. From the resulting, 1 mL was taken and mixed with 1 mL of distilled water and 1 mL of 0.1% FeCl_3_. The mixture was incubated for 10 min at room temperature in darkness. Absorbance was measured at 700 nm using deionized water as blank. Higher absorbance values were indicative of a higher reducing power.

#### 2.3.6. Bioactive Compounds

##### Total Phenols

The measurement and quantification of total phenols was determined as suggested by Singleton and Rossi [21]; Singleton et al. [22]. We began by standardizing 0.5–1 g of ground seeds with 5 mL of methanol and 2.5 mL of 1% NaCl solution. The reaction mixture consisted in placing 750 μL of 2% Na_2_CO_3_, 250 μL of 50% Folin-Ciocalteau reagent and 1375 μL of deionized water in a test tube, adding 250 μL of extract. The mixture was incubated at room temperature for 60 min, after which the measurement was done against a gallic acid (10–100 μg/mL) pattern curve at 725 nm absorbance. Results are shown in mg GA/g^−1^ dry weight (dw).

##### Flavonoids

Flavonoids from the dry material were extracted in methanol. An amount of 0.5 g of ground material from each bean variety was standardized with 5 mL of methanol, being centrifuged at 1138× *g* for 10 min at 4 °C. The mixture consisted in transferring 250 μL of the aliquot in a test tube, adding 75 μL of NaNO_2_, mixing using a vortex stirrer and letting it rest for 5 min. Then, 150 μL of aluminum chloride (AlCl) were added, after which 500 μL of NaOH were added, diluting to a final volume of 2.025 mL with water. Absorbance was measured by spectrophotometry at 510 nm. Results are shown in mg equivalent to catechin per gram per sample, based on the dry weight (mg CE/g^−1^ dw).

##### Anthocyanins

This determination used the pH differential method described by Wrolstad [23]. Anthocyanins from the dry material were extracted in methanol. An amount of 0.5 g of the ground bean material was standardized in 5 mL of methanol. The mixture was centrifuged at 1138× *g* for 10 min at 4 °C. The reaction mixture consisted of 2 phases. Phase 1 absorbance was measured by spectrophotometry at 460 nm, while phase 2 absorbance was measured at 710 nm. Results are reported as mg of cyanidin-3-glucoside (mg C3G/g^−1^ dw).

#### 2.3.7. Statistical analysis

Data obtained were subject to a variance and means separation analysis by means of the Tukey and correlation test, using a statistical program SAS [24] (SAS Institute, Inc., Cary, NC, USA). The Tukey test (95%) was used to determine the difference between the means of the bean varieties studied [25]. The significance levels of both tests are expressed as follows: * *p* < 0.05; ** *p* < 0.01; *** *p* < 0.001 and NS, not significant.

## 3. Results and Discussion

### 3.1. Physical Characteristics

#### 3.1.1. Grain Color

Grain color was determined according to the luminance (L*), chromaticity coordinates (a*, b*), the relative color purity (chroma) and the Hue angle. With respect to the luminance present in each bean variety studied, results obtained ranged from 27.55% to 81.84% in L*, where patola (a white colored bean) showed the highest luminance values at 81.84%, while black beans showed the lowest luminance values at 27.55% in L* (Table 2). Previous studies performed with wild varieties of Brazilian bean resulted in a range between 19.29 and 52.20% for such parameter [2]. These values are indicative of the luminous aspect studied in grains, given that, the darker the color, the lower the luminance. Thus, the varieties having a lighter colored seed coat showed higher luminance values, as is the case of the following varieties. Patola, navy bean, frijola and bayo, which have the lightest hues as compared to the remaining varieties studied. On the other hand, the black bean, reata bean, flor de mayo and flor de junio varieties showed the lowest luminance values, since they have a darker colored seed coat (Table 2). In Mexico there is a regional trend regarding consumption preferences, where yellow varieties are mainly consumed in the northwest, almost all colors varieties are consumed in the center, and dark and black varieties are consumed in the southeast [26]. 

With regard to chromaticity coordinates (a*, b*), results showed both negative (−0.006) and positive (14.68) results for a*. The negative value pertains to the canary bean variety, while the highest positive value obtained was seen in the flor de mayo variety (see Table 2). It is worth noting that within the CIELAB (L*, a*, b*) chromatic model, the positive values surrounding the letter a* indicate the presence of red hues, while the same letter having a negative value indicates the presence of greenish-yellowish hues. These results clearly match the chromatic model values, given that the canary bean variety, which obtained a negative value for a* (−a*) showed a yellowish hue with somewhat greenish shades. The flor de mayo variety, on the other hand, showed a reddish-purple hue, fully matching the chromatic model.

In the case of b*, results found also showed values ranging from −2.18 to 30.84, where the negative value pertains to the black bean variety, while the highest positive value was shown by the canary bean variety (see Table 2). It is worth noting that within the CIELAB chromatic model, the negative values associated with letter b* indicate the presence of blue hues, while the same letter having a positive value indicates the presence of yellowish hues. The results found in the bean varieties studied also match the chromatic model values, given that the canary bean variety, which had a positive b* value, showed a yellowish hue with greenish shades. Particularly, the black bean variety showed a bright black-bluish hue, which fully matches the chromatic model described earlier.

That being said, the relative purity of color, also known as chroma or color key, was found within the range of 2.31 to 30.84 chroma. The minimum value was shown by the black bean variety, while the maximum value was shown by the canary bean variety. Since the possible values range from 0 to 100, where 0 is indicative of low saturation and 100 is indicative of high saturation. Therefore, the lower the saturation of a given color, the greater the grayish hue and discoloration it will show. This was confirmed in this study, where the black bean variety obtained a low saturation (2.31), which indicates that the color of its seed coat has a dark bright shade within the gray-black scale, while the canary bean variety showed a medium saturation due the color of its seed coat.

Regarding the shade, tint or hue, negative as well as positive values were obtained. The black bean variety had a value of −1.22° (hue), while the patola variety reported a value of 1.46° (hue) (see Table 2). Therefore, one can infer that the bean varieties studied lie within a reasonable range, since hue degrees represent one of the basic properties or qualities within a color’s properties, where a stimulus can be described as similar or different (e.g. red, yellow, green or blue). It is important the determination of these three parameters, since these previous studies showed the possibility of selecting common varieties of bean with a grain color that is the most accepted between consumers [27]. On the other hand, Pérez Herrera et al. [28] and Iniestra-González et al. [29] mention that a dark colored seed coat could be indicative of a good antioxidant activity.

#### 3.1.2. Length, Width, Thickness and Weight

Common bean (*Phaseolus vulgaris* L.) is a highly varied common grain with regard to its physical characteristics, i.e. length, width, thickness and weight. Table 3 lists the results obtained for these characteristics, which were analyzed in all eleven bean varieties coming from the state of Zacatecas.

The dimensions observed with regard to grain length are quite similar in nine of the eleven bean varieties studied; however, there are significant differences with regard to the length in the patola and black bean varieties. Regarding this parameter, the patola variety has the longest length (18.03 mm), while the black bean variety has the shortest length (10.70 mm) (Table 3). 

With respect to grain width, differences observed are small though quite significant, given that the varieties studies showed very similar values, among which the following varieties stand out: Flor de junio and patola, which had a width of 7.60 mm and 11.06 mm, respectively (Table 3).

Speaking about thickness, this parameter was not the exception, having shown similar values among bean varieties. The patola variety showed the greatest thickness at 7.30 mm, while the pinto Saltillo variety had the smallest thickness at 4.83 (Table 3). It is worth noting that the difference in thickness in these two varieties is 2.47 mm. This difference does not seem too big, although it becomes totally evident when visually comparing these varieties.

An additional characteristic is grain weight. As expected, weight was influenced by the above-described parameters. There were significant differences in grain weight among bean varieties, which ranged from 28.86 to 83.72 g (for 100 grains) for the flor de junio and patola varieties, respectively (Table 3). Previous studies performed with varieties of Brazilian bean showed a lower weight ranged from 20.33 and 30.72 g for each 100 grains [30]. This demonstrates that varieties assessed in the current study are larger in size; therefore, they have a higher performance. This is a feature of interest in biofortification studies with microelements [31].

### 3.2. Physico-Chemical Analysis

With regard to fiber concentration, values ranged from 2.71% to 3.64%, where the flor de mayo variety stood out as having the highest concentration at 3.64%, while the bayo variety showed the lowest concentration at 2.71% (Table 4). A similar study conducted by Aguirre-Santos and Gomez-Aldapa [11] showed that fiber concentration ranged from 1.35% to 2.77%, which indicates that the values we obtained were greater to those identified by said authors. In our study, greater concentrations were obtained per variety. Worth noting is the fact that the variety showing the lowest concentration in our study, that is, bayo beans (2.71%) was within the highest concentration value (2.77%) of all varieties studied in the above-mentioned study.

Moreover, protein content ranged from 19.20% to 26.88%, where the reata bean variety showed the highest concentration (26.88%), while the flor de junio variety showed the lowest concentration (19.20%) (Table 4). According to Ulloa [32], the protein values seen in beans range from 14% to 33%. The varieties analyzed in our study lie within this range.

The values obtained for carbohydrates showed that the black bean variety has the highest carbohydrate concentration (65.79%), while the reata bean variety has the lowest carbohydrate concentration (57.16%) (Table 4). With regard to the investigations conducted by Campos-Vega [30] and Gómez-Aldapa [33], where carbohydrate concentrations ranged from 51.51% to 56.28%, it is worth noting that the bean varieties in our study significantly exceeded the values obtained by said authors. In our study, the bean variety having the lowest carbohydrate concentration, namely, the reata bean, exceeded the result obtained by the variety having the highest carbohydrate concentration by 0.88% in the above-mentioned study as indicated by the results of said study.

With regard to fat, it was found in small quantities. The black bean variety showed the lowest fat content at 0.78%, while the frijola variety showed the highest fat content at 2.19% (see Table 4). A similar study by Lijiao Kan [34] reported that fat content ranged from 1.05% to 2.83%. These levels exceed the minimum content observed in our study but lie within the maximum level observed.

With respect to the energy content present in the bean varieties studied, the following concentrations were found: The highest energy content (355.15 kcal) was seen in the frijola bean variety, while the lowest energy content (346.96 kcal) was seen in the canary bean variety (Table 4).

With regard to ash and moisture, significant differences were identified among the bean varieties studied. Speaking about ash, patola was the most outstanding variety, having shown a 4.72% increase, while the flor de junio variety showed a 3.89% increase. When it comes to moistures, the canary bean variety stood out at 7.42%, while the Japanese bean variety only showed a 6.14% level of moisture (Table 4). According to investigations conducted by Campos-Vega [33] the moisture percentage ranges from 8.0% up to 11.95%. In our study, the variety Canary bean contains a lower percentage (7.42%) than the lowest value reported by said authors.

### 3.3. Mineral Analysis

#### 3.3.1. Microelements

Results obtained from the micronutrient analysis show significant differences in the bean varieties studied. The values obtained are shown in Table 5. This chart shows the copper, nickel, manganese, iron and zinc concentrations founds in bean grains.

The iron levels found in the bean varieties in this study lie within the range of 21.62 mg/kg (Japanese variety) to 105.29 mg/kg (Canary bean). According to Acosta-Gallegos et al. [35], who conducted a study on Mexican bean varieties, their study revealed that the iron concentration in beans ranged from 24.8 mg/kg to 57.5 mg/kg. The variety of Canary bean contains 83.11% more iron than the higher variety reported by Acosta-Gallegos et al. [35]. Iron content is one of the most important parameters in biofortification studies, since approximately 60% of world population have this microelement deficiency [36].

The zinc concentrations shown by the varieties studied lie within the range of 6.74 mg/kg to 48.18 mg/kg. These values correspond to the Japanese bean and Bayo varieties, respectively. These results are similar to those found by Acosta-Gallegos et al. [35], who determined the zinc content found in a number of bean varieties from different parts of Mexico. The values reported in said study ranged from 27.1 mg/kg to 41.3 mg/kg. Bayo bean contains 6.88 mg/kg more zinc than the higher zinc variety analyzed in the study of Acosta-Gallegos et al [35]. Previous studies performed by Guzmán-Maldonado et al. [37] show a mean of 17 mg/kg of zinc in bean varieties of Jalisco and Durango. On the other hand, values between 24–38 mg/kg were reported by Koehler et al. [38] reported. 

Copper content varied from 8.34 mg/kg to 13.24 mg/kg, where bayo beans showed the highest concentration among all eleven varieties used in the study (13.24 mg/kg), while black beans showed the lowest concentration (8.34 mg/kg).

With regard to the content of nickel found in the bean varieties studied, the following concentrations were obtained: The highest nickel concentration (10.40 mg/kg) was seen in the Japanese bean variety, while the lowest nickel concentration (2.03 mg/kg) was identified in the bayo bean variety. The manganese concentrations found revealed that values range from 5.41 mg/kg to 38.54 mg/kg, where the black bean variety showed the highest zinc concentration (38.54 mg/kg), while the pinto Saltillo variety showed the lowest concentration (5.41 mg/kg).

#### 3.3.2. Macroelements

Results obtained regarding the content of macronutrients showed significant differences. The values obtained are shown in Table 6, which shows the concentration of calcium, sodium, magnesium, potassium, phosphorus and nitrogen found in each bean variety used in the study.

Calcium content results showed the following concentrations: the highest calcium concentration (0.29%) was seen in the navy bean variety, while the lowest calcium concentration (0.12%) was observed in the Patola variety (see Table 6). Previous investigations showed very similar values to those obtained in our study. For instance, Acosta-Gallegos et al. [35] reported that the calcium content found in bean grains ranges from 0.11% to 0.63%. Hence, the bean varieties used in our study lie within this range. As described at the beginning of this paragraph, our bean varieties showed results in the range of 0.12% to 0.29% in calcium content, which indicates that our bean varieties lie within the normal range established for this macronutrient.

With regard to sodium content, concentrations found are minimal and are very similar among different bean varieties. These small concentrations showed a mean of 0.003%, given that the sodium content values obtained ranged from 0.002% to 0.004%. The lowest sodium concentration was seen in the flor de mayo and patola varieties (0.002%), while the frijola, canary bean, bayo and pinto Saltillo varieties showed the highest concentration (0.004%). Consequently, the mean concentration (0.003%) was identified in the remaining varieties.

The magnesium content found is within the range from 0.02% to 0.16%, where the bayo and flor de junio varieties showed the highest magnesium concentration at merely 0.02%, while the Japanese bean variety showed the highest magnesium concentration at 0.16% (see Table 6). 

The values obtained for potassium content show that the canary bean variety has the lowest content (only 0.41%), whereas the bayo and Japanese bean varieties showed the highest content (1.32%). A similar study conducted by Sánchez et al. [16] shows that the potassium levels reported (1.63% to 4.65%) are higher than those obtained in our study since the levels found in the bean varieties used in our study range from 0.41% to 1.32%. The differences in values obtained are deemed to be the result of agroclimatic conditions, crop management and genetic varieties. 

With regard to phosphorus and nitrogen concentrations, significant differences were identified among the bean varieties studied. For phosphorus, the values obtained range from 0.13% to 0.40%, where the frijola variety stands out as having the highest phosphorus concentration at 0.40%, while the navy bean and bayo varieties show the lowest concentration at 0.13% (Table 6). With regard to the nitrogen concentration, values obtained range from 3.07% to 4.30%, where the reata bean variety stands out as having the highest concentration (4.30%), whereas the flor de junio variety showed the lowest concentration (only 3.07%) (see Table 6). A study conducted by Sánchez et al. [16] indicates that the highest phosphorus concentration found in bean grains is 1.14% and that the nitrogen concentration ranges from 2.75% to 4.75%. The bean varieties used in our study showed lower phosphorus concentrations than those found by the above-mentioned author, although the nitrogen concentrations identified in our study are quite similar.

### 3.4. Antioxidant Capacity

**Figure 1:**
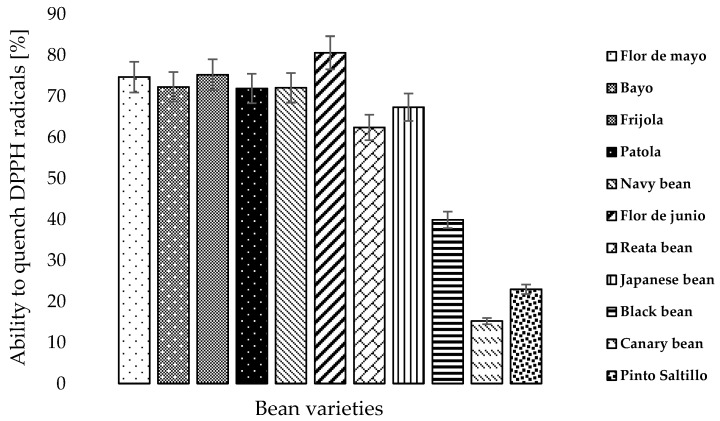
Antioxidant capacity (DPPH) of bean varieties grown in the state of Zacatecas, Mexico. Data are shown as the mean ± standard error (*n* = 3). DPPH, 2-diphenyl-1 picryl hydrazyl.

Figure 1 shows the antioxidant capacity values of the bean varieties grown and consumed in the state of Zacatecas. The eleven bean varieties studied were classified in three major groups: (1) High level, (2) Medium level and (3) Low level of antioxidant capacity. Statistically, all assessed varieties have an activity very similar. The flor de junio, frijola, flor de mayo, bayo, navy bean and patola varieties showed a high level of antioxidant capacity. These varieties share certain physical characteristics. Navy bean and patola varieties are both white, the bayo and frijola varieties share the same brown color and the same size, and the flor de junio and the flor de mayo varieties are characterized by an odd purple color. From these, the flor de junio variety stands out as having the highest antioxidant capacity (80.62% ability to quench DPPH radicals). The Japanese bean and reata bean varieties showed a medium antioxidant capacity, where the Japanese bean stood out among the medium level category, having obtained a 67.33% inhibition capacity. A biofortification study with green beans was performed by Sida-Arreola et al. [39] and values ranged from 45–80% inhibition were reported. The varieties assessed in our study show similar results, considered high for bean varieties that have not been biofortified. Therefore, it is feasible to perform biofortification studies with these bean varieties to increase their antioxidant capacity and thus, to improve the consumer health.

Meanwhile, the Black bean, Canary bean and Pinto Saltillo varieties showed a low level of antioxidant capacity. These varieties possess different physical characteristics when it comes to color. For example, the canary bean variety is yellow, while the pinto Saltillo variety is brown (with a speckled pattern) and the black bean variety is characterized by dark shades. In spite of having very different seed coat colors, these varieties have very similar in size. The canary bean variety showed the lowest level of antioxidant activity (15.23% inhibition).

Among the eleven bean varieties studied, the Flor de junio variety stands out as having the highest antioxidant capacity (80.62%), compared to the canary bean variety, which showed the lowest antioxidant capacity (15.23%). There was an 81.11% increase between the bean varieties showing the highest and lowest antioxidant capacity. It should be noted that these two varieties showed the same behavior regarding the reducing power, where Flor de Junio stands out as the most outstanding variety.

### 3.5. Reducing Power

**Figure 2:**
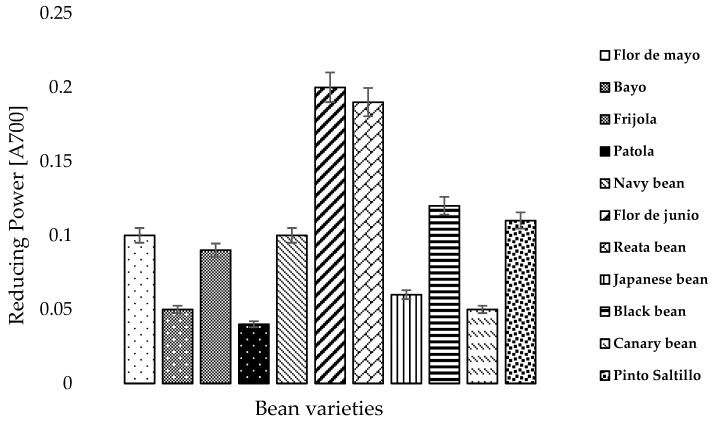
Reducing power of bean varieties grown in the state of Zacatecas, Mexico, classified in three major groups: (1) High level, (2) Medium level and (3) Low level. Data are shown as the mean ± standard error (*n* = 3).

Figure 2 shows the reducing power values for bean varieties grown in the state of Zacatecas. The eleven bean varieties were classified in three major groups: (1) High level, (2) Medium level and (3) Low level reducing power. The high level group included the following bean varieties: The flor de junio and reata bean varieties, which share several characteristics, such as their purple color and similar size. The flor de junio variety stands out as having the highest reducing power (0.2%). The second group is comprised by the vast majority of varieties studied, which are the following: Black bean, pinto Saltillo, frijola, flor de mayo and navy bean. The black bean variety stood out in this group, having shown a reducing power of 0.12%. Differences in the reducing power of the assessed varieties were statistically equal.

The third group is comprised by the following bean varieties: Japanese bean, canary bean, patola and bayo, where the patola variety had the lowest reducing power. Hence, the flor de junio variety stood out as having the highest reducing power concentration at 0.2%, while the patola variety showed the lowest reducing power concentration at 0.04%. This resulted in an 80% increase between the varieties having the highest and lowest reducing power concentration. Results of the study performed with varieties of mungo bean showed a range between 0.002% and 1.96% [40]. Our results are within this range and found over the mean obtained in the study mentioned above.

### 3.6. Bioactive Compounds

#### 3.6.1. Total Phenols

With regard to the total phenols content, the values identified ranged from 46.75 to 114.29 mg GA/g^−1^ dw, where the frijola variety showed the highest concentration at 114.29 mg GA/g^−1^ dw, while the navy bean variety showed the lowest concentration at 46.75 gallic acid/g extract, (see Table 7). A previous study done by Gracia-Nava [41], who performed a quantification in total phenols, revealed results showing that phenol concentration ranges from 19.75 mg to 221.48 mg GA/g^−1^ dw. These values match the value range observed in our study.

#### 3.6.2. Flavonoids

With regard to flavonoids, results (Table 7) show that flavonoid concentrations in all bean varieties contain are much lower than the total phenol concentrations reported. This was expectable, given that flavonoids are a subgroup deriving from phenolic compounds. A similar study conducted by Gracia-Nava [39] reported a minimum value of 2.26 mg Cat/g^−1^ dw, whereas the maximum value reported was 25.94 mg Cat/g^−1^ dw. However, the values obtained in our study range from 0.33 to 2.18 mg Cat/g^−1^ dw, which correspond to the black bean and patola varieties, respectively.

#### 3.6.3. Anthocyanins

With respect to anthocyanins content, values obtained indicate that the reata bean variety showed the lowest anthocyanins content (only 0.20 mg EC3G/g bean flour), while the navy bean variety showed the highest content (2.57 mg EC3G/g bean flour). In a study conducted by Reynoso-Camacho et al. [34], levels of anthocyanins were reported to be 3.75 mg EC3G/g bean flour. When comparing his study to ours, we noticed that the bean variety showing the highest anthocyanins content in our study has a lower concentration, although such content is still considerably good.

The varieties showing the most remarkable individual results are as follows: Canary beans had the highest iron content (105.29 mg/kg), while bayo beans had the highest zinc concentration (48.18 mg/kg) and reata beans had the highest level of protein (26.88%). On the other hand, the varieties showing the most remarkable overall results with regard to zinc, iron and protein content and antioxidant capacity are as follows: Flor de junio, flor de mayo, reata bean, navy bean and pinto Saltillo. Flor de mayo: This is a highly complete bean variety, given that it stands out as a result of its reducing power (0.10%), antioxidant capacity (74.68% inhibition), and concentration of potassium (1.04%), magnesium (0.13%), nickel (8.89 mg/kg), manganese (25.45 mg/kg), zinc (31.00 mg/kg), fiber (3.64%), flavonoids (1.70 mg Cat/g^−1^ dw, and anthocyanins (0.62 mg EC3G/g bean flour). Reata bean: This variety has a high content of nitrogen (4.30%) and, consequently, a high protein concentration (26.88%). It also has good concentration level of phosphorus (0.18%), magnesium (0.10%), copper (11.19 mg/kg), nickel (7.54 mg/kg), energy (351.73 kcal), phenols (94.35 mg GA/g^−1^ dw) and flavonoids (1.20 mg Cat/g^−1^ dw). The navy bean variety shows a good concentration level of reducing power (0.10%), antioxidant capacity (72.06% inhibition), nitrogen (3.70%), potassium (1.08%), calcium (0.29%), copper (12.38 mg/kg), manganese (30.60 mg/kg), protein (23.16%), and fiber (3.24%), as well as a high anthocyanins content (2.57 mg EC3G/g bean flour). The pinto Saltillo bean variety has an extensive concentration of reducing power (0.11%), nitrogen (4.12%), magnesium (0.11%), calcium (0.25%), copper (11.82 mg/kg), zinc (25.96 mg/kg), protein (25.78%), fiber (3.44%), phenols (90.16 mg GA/g^−1^ dw) and anthocyanins (mg EC3G/g bean flour). Nonetheless, the Flor de junio variety proved to be the most outstanding and complete bean variety, having a high concentration of antioxidant capacity (80.62% inhibition) and reducing power (0.20%). These parameters are affected by the concentration of phenols (94.82 mg GA/g^−1^ dw) and flavonoids (1.30 mg Cat/g^−1^ dw). Likewise, it shows good levels when it comes to iron (90.97 mg/kg), zinc (27.23 mg/kg), fiber (3.22%), energy (353.50 kcal), calcium (0.24%) and phosphorus (0.17%). Due to their concentration of minerals, bioactive compounds and antioxidant capacity, these varieties have been considered for inclusion in a Mexico-based biofortification program using micronutrients aimed at counteracting malnutrition and promoting food security nationwide and also in the international arena.

## 4. Conclusions

The varieties showing the most remarkable individual results are as follows: Canary beans had the highest iron content (105.29 mg/kg), while bayo beans had the highest zinc concentration (48.18 mg/kg) and reata beans had the highest level of protein (26.88%). The varieties showing the most remarkable results with regard to zinc, iron and protein content and antioxidant capacity were as follows: Flor de junio, flor de mayo, reata beans, navy beans and pinto Saltillo; however, the most remarkable variety in comprehensive terms was flor de junio, which showed a reducing power of 0.20%, an antioxidant capacity of 80.62% inhibition, a protein content of 0.17%, in addition to Ca (0.24%), Fe (90.97 mg/kg), Zn (27.23 mg/kg), fiber (3.22%), energy (353.50 kcal), phenols (94.82 mg GA/g^−1^ dw) and flavonoids (1.30 mg Cat/g^−1^ dw). Due to their concentration of minerals, bioactive compounds, and antioxidant capacity, these varieties have been considered for inclusion in a Mexico-based biofortification program using micronutrients aimed at counteracting malnutrition and promoting food security nationwide and even worldwide.

## Figures and Tables

**Table 1:** Bean varieties selected in the state of Zacatecas to be studied.

Bean Variety	Origin	Date Obtained	Picture
Flor de mayo	Calera, Zacatecas	4 June 2018	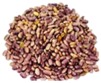
Bayo	Calera, Zacatecas	4 June 2018	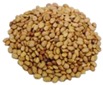
Frijola	Calera, Zacatecas	4 June 2018	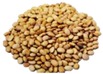
Patola	Calera, Zacatecas	4 June 2018	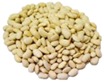
Navy bean	Calera, Zacatecas	4 June 2018	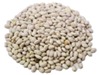
Flor de junio	Calera, Zacatecas	4 June 2018	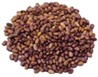
Reata bean	Calera, Zacatecas	4 June 2018	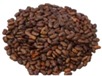
Japanese bean	Calera, Zacatecas	4 June 2018	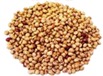
Black bean	Calera, Zacatecas	4 June 2018	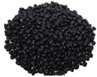
Canary bean	Calera, Zacatecas	4 June 2018	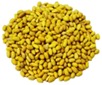
Pinto saltillo	Calera, Zacatecas	4 June 2018	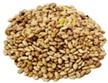

**Table 2:** Grain color of bean varieties grown in the state of Zacatecas, Mexico.

Bean Variety	L*	a*	b*	Chroma	Hue
Flor de mayo	53.13	**14.68**	7.60	16.54	0.47
Bayo	65.44	7.11	20.01	21.23	1.22
Frijola	66.75	6.41	19.58	20.60	1.25
Patola	**81.84**	1.55	13.99	14.05	**1.46**
Navy bean	79.54	2.05	10.31	10.51	1.37
Flor de junio	52.04	14.19	9.13	16.90	0.57
Reata bean	46.86	13.08	8.87	15.82	0.59
Japanese bean	55.98	11.57	15.34	19.22	0.92
Black bean	27.55	0.78	−2.18	2.31	−1.22
Canary bean	67.63	−0.006	**30.84**	**30.84**	−0.52
Pinto Saltillo	65.06	5.71	13.54	14.69	1.17
Significance	***	***	***	***	***
LSD	4.8888	1.3998	1.9912	1.813	1.6055

Significance levels: * *p* < 0.05, ** *p* < 0.01, *** *p* < 0.001, NS, not significant. Note: numbers in **bold** indicate the highest values, while underlined numbers indicate the lowest values.

**Table 3:** Biodiversity of bean varieties grown in the state of Zacatecas, Mexico (physical characteristics).

Bean Variety	Length (mm)	Width (mm)	Thickness (mm)	Weight of 100 Grains (g)
Flor de mayo	13.26	7.73	5.66	29.49
Bayo	12.03	9.10	5.83	35.26
Frijola	13.73	9.63	6.03	47.80
Patola	**18.03**	**11.06**	**7.30**	**83.72**
Navy bean	12.26	7.96	6.43	34.31
Flor de junio	11.40	7.60	6.00	28.86
Reata bean	16.23	8.66	6.30	47.94
Japanese bean	11.46	9.06	7.23	40.98
Black bean	10.70	7.76	5.40	30.75
Canary bean	12.86	7.63	6.43	45.01
Pinto Saltillo	12.60	8.00	4.83	32.70
Significance	NS	NS	NS	NS
MSD	1.8275	1.2914	1.6455	3.6038

Significance levels: * *p* < 0.05, ** *p* < 0.01, *** *p* < 0.001, NS, not significant. Note: numbers in **bold** indicate the highest values, while underlined numbers indicate the lowest values.

**Table 4:** Physico-chemical composition of the different bean varieties grown and consumed in the state of Zacatecas, Mexico.

Bean Variety	Ash	Fat	Moisture	Fiber	Carbohydrates	Protein	Energy
	(%)	(%)	(%)	(%)	(%)	(%)	(kcal)
Flor de mayo	4.14	1.03	6.36	**3.64**	63.47	21.36	348.59
Bayo	4.15	1.39	7.12	2.71	62.53	22.10	351.03
Frijola	4.41	**2.19**	6.72	2.82	59.57	24.29	**355.15**
Patola	**4.72**	2.09	7.17	3.48	60.60	21.94	348.97
Navy bean	4.24	1.11	6.46	3.24	61.79	23.16	349.79
Flor de junio	3.89	1.74	6.69	3.22	65.26	19.20	353.50
Reata bean	4.40	1.73	6.66	3.17	57.16	**26.88**	351.73
Japanese bean	4.42	0.97	6.14	2.88	62.67	22.92	351.09
Black bean	4.12	0.78	6.35	2.82	**65.79**	20.14	350.74
Canary bean	4.26	1.08	**7.42**	2.93	59.30	25.01	346.96
Pinto Saltillo	4.29	1.11	6.73	3.44	58.65	25.78	347.71
Significance	***	***	***	***	***	***	***
MSD	0.0515	0.0763	0.0545	0.0832	0.2659	0.05	0.3854

Significance levels: * *p* < 0.05, ** *p* < 0.01, *** *p* < 0.001, NS, not significant. Note: numbers in **bold** indicate the highest values, while underlined numbers indicate the lowest values.

**Table 5:** Concentration of micronutrients (mg/kg) found in bean varieties grown and consumed in the state of Zacatecas, Mexico.

Bean Variety	Copper	Nickel	Manganese	Iron	Zinc
Flor de mayo	10.15	8.98	25.45	55.78	31.00
Bayo	**13.24**	2.03	10.07	76.48	**48.18**
Frijola	10.14	7.89	12.16	97.76	8.52
Patola	9.85	10.12	25.42	65.74	7.09
Navy bean	12.38	4.89	30.60	53.90	18.12
Flor de junio	8.95	2.78	12.86	90.97	27.23
Reata bean	11.19	7.54	6.07	65.57	17.09
Japanese bean	12.76	**10.40**	15.90	21.62	6.74
Black bean	8.34	5.64	**38.54**	84.59	13.11
Canary bean	10.72	3.29	28.48	**105.29**	27.20
Pinto Saltillo	11.82	6.04	5.41	62.48	25.96
Significance	NS	***	***x	***	***
MSD	5.7385	5.1612	25.183	46.342	28.372

Significance levels: * *p* < 0.05, ** *p* < 0.01, *** *p* < 0.001, NS, not significant. Note: numbers in **bold** indicate the highest values, while underlined numbers indicate the lowest values.

**Table 6:** Concentration of macronutrients (%) found in bean varieties grown and consumed in the state of Zacatecas, Mexico.

Bean Ariety	Nitrogen	Phosphorus	Potassium	Magnesium	Sodium	Calcium
Flor de mayo	3.41	0.16	1.04	0.13	0.002	0.14
Bayo	3.53	0.13	1.32	0.02	**0.004**	0.17
Frijola	3.88	**0.40**	0.74	0.08	**0.004**	0.22
Patola	3.51	0.18	0.78	**0.16**	0.002	0.12
Navy bean	3.70	0.13	1.08	0.08	0.003	**0.29**
Flor de junio	3.07	0.17	0.43	0.02	0.003	0.24
Reata bean	**4.30**	0.18	0.63	0.10	0.003	0.25
Japanese bean	3.66	0.19	**1.32**	**0.16**	0.003	0.21
Black bean	3.22	0.14	1.25	0.08	0.003	0.25
Canary bean	4.00	0.16	0.41	0.05	**0.004**	0.14
Pinto Saltillo	4.12	0.16	0.86	0.11	**0.004**	0.25
Significance	**	**	**	*	***	*
MSD	0.008	0.0647	0.7354	0.1421	0.0017	0.1026

Significance levels: * *p* < 0.05, ** *p* < 0.01, *** *p* < 0.001, NS, not significant. Note: numbers in **bold** indicate the highest values, while underlined numbers indicate the lowest values.

**Table 7:** Concentration of bioactive compounds in bean varieties grown and consumed in the state of Zacatecas, Mexico.

Bean Variety	Total Phenols (mg gallic acid/g^−1^ dw)	Flavonoids (mg catechin/g^−1^ dw)	Anthocyanins (mgEC3G/g^−1^ dw)
Flor de mayo	83.58	1.70	0.62
Bayo	89.83	1.10	0.50
Frijola	**114.29**	1.16	0.27
Patola	59.92	**2.18**	0.33
Navy bean	46.75	0.35	**2.57**
Flor de junio	94.82	1.30	0.44
Reata bean	94.35	1.20	0.20
Japanese bean	101.50	0.97	0.70
Black bean	80.33	0.33	0.43
Canary bean	68.01	0.46	1.16
Pinto Saltillo	90.16	0.82	0.59
Significance	*	***	***
MSD	65.988	0.6481	0.659

Significance levels: * *p* < 0.05, **p < 0.01, ***p < 0.001, NS, not significant. Note: numbers in **bold** indicate the highest values, while underlined numbers indicate the lowest values.
